# Effects of Advanced Trauma Life Support® training compared with standard care on adult trauma patient outcomes (ADVANCE TRAUMA): study protocol for a stepped-wedge cluster randomised trial

**DOI:** 10.1186/s13063-026-09491-z

**Published:** 2026-02-03

**Authors:** Samriddhi Ranjan, Sara Fälth, Prashant Kharat, Abhinav Bassi, Girish D. Bakhshi, Debojit Basak, Johanna Berg, Shamita Chatterjee, Li Felländer-Tsai, Karla Hemming, Vivekanand Jha, Jessica Kasza, Monty Khajanchi, James Martin, Anurag Mishra, Anna Olofsson, Nobhojit Roy, Rajdeep Singh, Kapil Dev Soni, Martin Gerdin Wärnberg

**Affiliations:** 1 The George Institute for Global Health, New Delhi, India; 2 Department of Global Public Health, Karolinska Institutet, Stockholm, Sweden; 3 Department of General Surgery, Grant Medical College and Sir Jamshedjee Jeejeebhoy Group of Hospitals, Mumbai, India; 4 Department of General Surgery, Institute of Post Graduate Medical Education and Research, Kolkata, India; 5 Emergency Medicine, Department of Internal and Emergency Medicine, Skåne University Hospital, Malmö, Sweden; 6 Department of Clinical Science, Intervention and Technology, Division of Orthopedics and Biotechnology, Karolinska Institutet, Stockholm, Sweden; 7 Department of Trauma, Acute Surgery and Orthopaedics, Karolinska University Hospital, Stockholm, Sweden; 8 Department of Applied Health Science, University of Birmingham, Birmingham, UK; 9 School of Public Health and Preventive Medicine, Monash University, Melbourne, Australia; 10 Department of Surgery, Seth G. S. Medical College and K.E.M. Hospital, Mumbai, India; 11 Department of General Surgery, Maulana Azad Medical College, New Delhi, India; 12 Institute of Environmental Medicine, Karolinska Institutet, Stockholm, Sweden; 13 Program for Global Surgery and Trauma, The George Institute for Global Health, New Delhi, India; 14 Critical and Intensive Care, JPN Apex Trauma Center, All India Institute of Medical Sciences, New Delhi, India; 15 Perioperative Medicine and Intensive Care, Karolinska University Hospital, Solna, Sweden

**Keywords:** Advanced Trauma Life Support, Traumatology, Life support care

## Abstract

**Background:**

Advanced Trauma Life Support® (ATLS®) is the most widely adopted form of trauma life support training worldwide, but there is no high-quality evidence that it can improve patient outcomes. The aim of this trial is to compare the effects of ATLS® training with standard care on outcomes in adult trauma patients.

**Methods:**

ADVANCE TRAUMA is a batched stepped-wedge cluster randomised controlled trial in India, where ATLS® is not routinely implemented. The trial will be conducted in 30 clusters (hospitals), organised into six batches of five clusters each. All clusters transition through three phases: first, a standard care phase; second, a 1-month transition phase, during which the training is delivered; and finally, an intervention phase, for a total of 13 months. Each cluster is randomised to an implementation sequence that defines the duration of the standard care and intervention phases. The trial will include at least 4320 adult trauma patients (≥ 15 years) who present to emergency departments and are subsequently admitted or transferred for admission. The primary outcome is in-hospital mortality within 30 days of arrival at the emergency department.

**Discussion:**

This will be the first large-scale trial to provide robust evidence of the effectiveness of ATLS® since the programme was initiated in 1978. Regardless of the findings, this study will have important implications for trauma life support training globally. If ATLS® training improves patient outcomes, ways to promote its use and optimise its implementation, especially in low- and middle-income countries such as India, should be explored. If patient outcomes do not improve, trauma life support training must change.

**Trial registration:**

Clinical Trials Registry–India (CTRI/2024/07/071336), ClinicalTrials.gov (NCT06321419, first registered 2024–03–20).

**Graphical Abstract:**

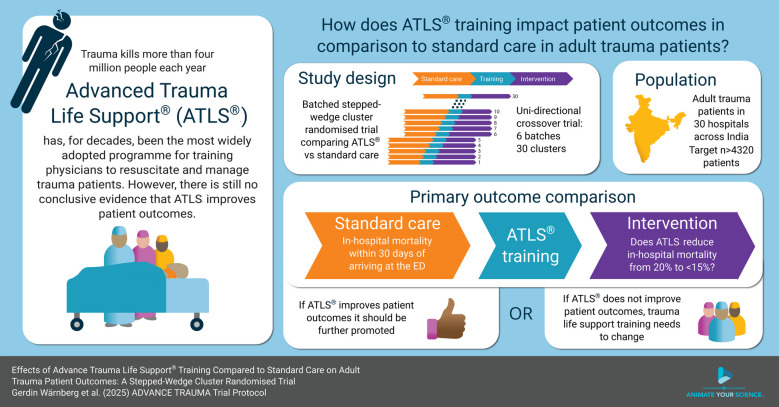

## Administrative information

**Table:** 

Title {1}	Effects of Advanced Trauma Life Support® Training Compared with Standard Care on Adult Trauma Patient Outcomes (ADVANCE TRAUMA): A Stepped-Wedge Cluster Randomised Trial
Trial registration {2a and 2b}	Clinical Trials Registry – India (CTRI/2024/07/071336), ClinicalTrials.gov (NCT06321419, 2024–03–20)
Protocol version {3}	1.5.0, 2025–06–12
Funding {4}	Swedish Research Council (reg. no. 2023–03128) and Laerdal Foundation (reg. no. 2023–0297)
Author details {5a}	Samriddhi Ranjan (SR) The George Institute for Global Health, New Delhi, India Sara Fälth (SF) Department of Global Public Health, Karolinska Institutet, Stockholm, Sweden Prashant Kharat (PK) The George Institute for Global Health, New Delhi, India Abhinav Bassi (AB) The George Institute for Global Health, New Delhi, India G D Bakhshi (GB) Department of General Surgery, Grant Medical College and Sir Jamshedjee Jeejeebhoy Group of Hospitals, Mumbai, India Debojit Basak (DB) Institute of Post Graduate Medical Education and Research, Kolkata, India Johanna Berg (JB) • Department of Global Public Health, Karolinska Institutet, Stockholm, Sweden • Emergency Medicine, Department of Internal and Emergency Medicine, Skåne University Hospital, Malmö, Sweden Shamita Chatterjee (SC) Department of General Surgery, Institute of Post Graduate Medical Education and Research, Kolkata, India Li Felländer-Tsai (LFT) • Department of Clinical Science, Intervention and Technology, Karolinska Institutet, Stockholm, Sweden • Department of Reconstructive Orthopedics, Karolinska University Hospital, Stockholm, Sweden Karla Hemming (KH) Institute of Applied Health Research, University of Birmingham, Birmingham, UK Vivekanand Jha (VJ) The George Institute for Global Health, New Delhi, India Jessica Kasza (JK) School of Public Health and Preventive Medicine, Monash University, Melbourne, Australia Monty Khajanchi (MK) Department of Surgery, Seth G. S. Medical College and K.E.M. Hospital, Mumbai, India James Martin (JM) Institute of Applied Health Research, University of Birmingham, Birmingham, UK Anurag Mishra (AM) Department of General Surgery, Maulana Azad Medical College, New Delhi, India Anna Olofsson (AO) Institute of Environmental Medicine, Karolinska Institutet, Stockholm, Sweden Nobhojit Roy (NR) • Program for Global Surgery and Trauma, The George Institute for Global Health, New Delhi, India • Department of Global Public Health, Karolinska Institutet, Stockholm, Sweden Rajdeep Singh (RS) Department of General Surgery, Maulana Azad Medical College, New Delhi, India Kapil Dev Soni (KDS) Critical and Intensive Care, JPN Apex Trauma Center, All India Institute of Medical Sciences, New Delhi, India Martin Gerdin Wärnberg (MGW) • Department of Global Public Health, Karolinska Institutet, Stockholm, Sweden • Perioperative Medicine and Intensive Care, Karolinska University Hospital, Solna, Sweden
Name and contact information for the trial sponsor {5b}	Karolinska Institutet, 171 77 Stockholm, Sweden Contact person: Martin Gerdin Wärnberg, martin.gerdin@ki.se
Role of sponsor {5c}	This is an investigator-initiated study. The sponsor contact person is also the principal investigator. The funding bodies had no role in the design of this trial, the preparation of it, nor the decision to submit this protocol for publication

## Introduction

### Background and rationale {6a}

Each year, 4.3 million people die from trauma [1]. Trauma is the leading cause of death related to poor health care quality [2], and most deaths from trauma occur within the first 24-48 h [3]. Trauma is also the greatest cause of disability adjusted life years among people aged 10–49 years [4]. Traumatic brain injury and exsanguination are the most common causes of trauma-related deaths [5, 6]. Most preventable trauma deaths from trauma are caused by errors in clinical judgement during initial resuscitation or early care, including airway management and haemorrhage control, even when the deaths occur later during the hospital stay [5, 7].

Several trauma life support training programmes have been developed to improve the early management of patients in hospitals by providing a structured framework for assessment and treatment [8–12]. The proprietary Advanced Trauma Life Support® (ATLS®) is the most established trauma life support training programme and more than one million physicians in more than 80 countries have been trained in the programme since the first course in 1978 [13]. In the USA and many other countries training in ATLS® is virtually mandatory for trauma care physicians [14]. However, uptake in lower-resourced settings has been slow, potentially due to high costs [10].

Three randomised studies have shown that ATLS® improves knowledge and clinical skills [15–17], but no randomised controlled trials or high-quality quasi-experimental studies have indicated that ATLS® improves patient outcomes [8, 9, 11, 12, 18]. We have conducted an updated systematic review [19], and estimated a pooled odds ratio of 0.51 (95% CI 0.37; 0.69) from ten heterogeneous (*I*^*2*^ 0.7) observational studies on the effect of ATLS®on mortality [20–29]. We also conducted a pilot study showing that a full-scale trial should be feasible [30, 31], as well as semi-structured interviews that indicated high acceptability of our research and helped to identify important outcomes [32].

### Objectives {7}

The aim of this trial is to compare the effects of ATLS® training with standard care on outcomes in adult trauma patients.

## Trial design {8}

**Fig. 1:**
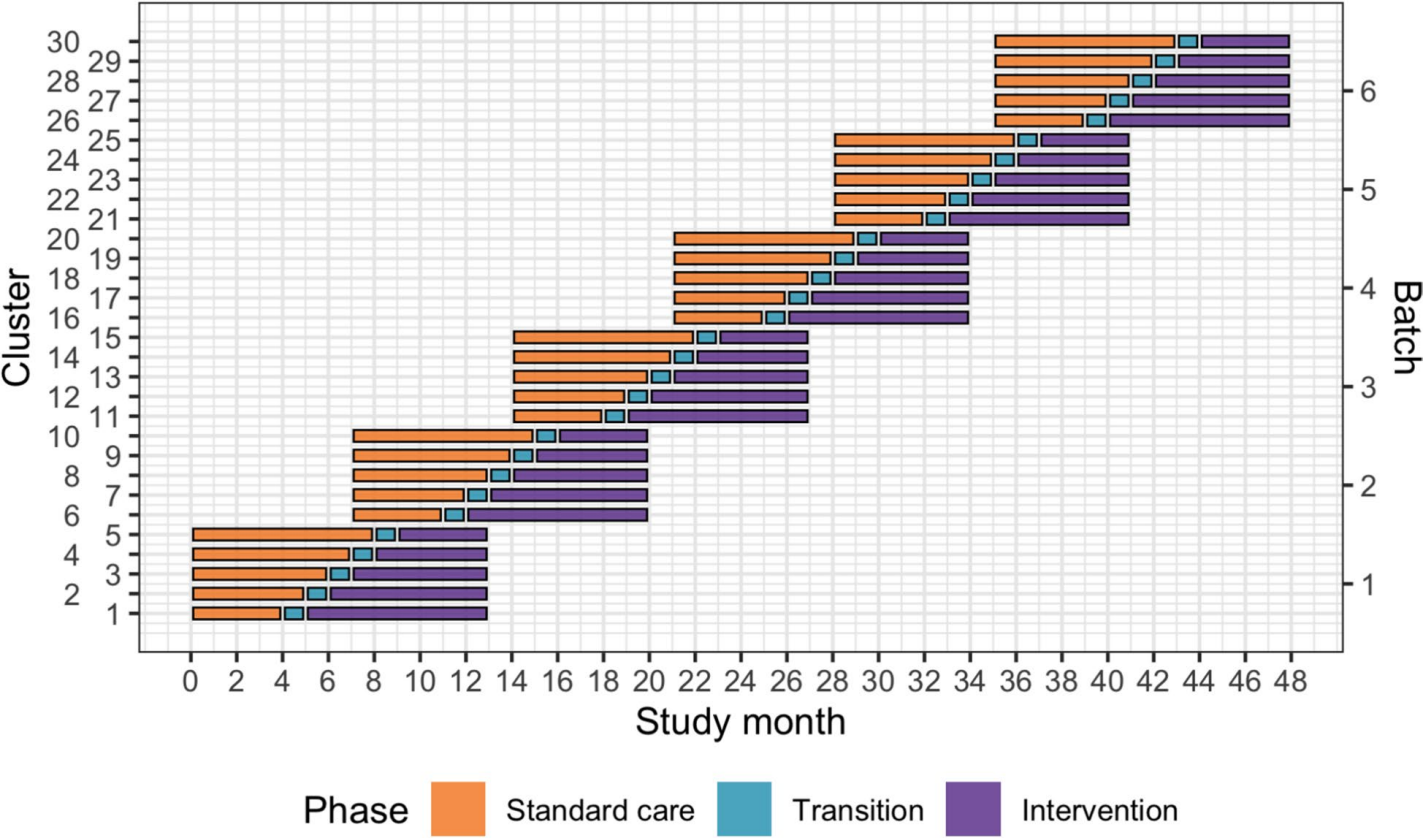
Trial design. Lines represent the duration of patient inclusion across clusters and phases. Clusters will be sequentially allocated to a batch based on when they enter the study. Within each batch, clusters will then be randomised to an intervention implementation sequence

This is a batched stepped-wedge cluster randomised controlled trial in India (see Fig. 1). The stepped-wedge trial is a unidirectional crossover trial, with a randomised time point when clusters cross over from standard care to the intervention [33]. In this trial, the unit of randomisation is the hospital: that is, the clusters randomised to the sequences of the stepped wedge design are the hospitals. Within each hospital, one or more units of physicians performing initial resuscitation of trauma patients in the emergency department may be trained. The number of units that will be trained in each hospital will depend on the sizes of these units and the volumes of patients the physicians attend. If more than one unit is trained in the same hospital, these are all considered part of the same cluster for the purpose of randomisation.

We will include a total of 30 clusters in six batches, with five clusters in each batch. The clusters in each batch will be randomised to one of five implementation sequences, with one cluster randomised to each implementation sequence. All clusters will transition through three phases: first, a standard care phase; second, a transition phase lasting 1 month during which the ATLS® training will be delivered to the physicians; and finally, the intervention phase. The period of participant inclusion within any cluster will last for a total of 13 months. The duration of the standard care and intervention phases will be determined by the implementation sequence. The anticipated overlap between batches is 6 months.

**Fig. 2:**
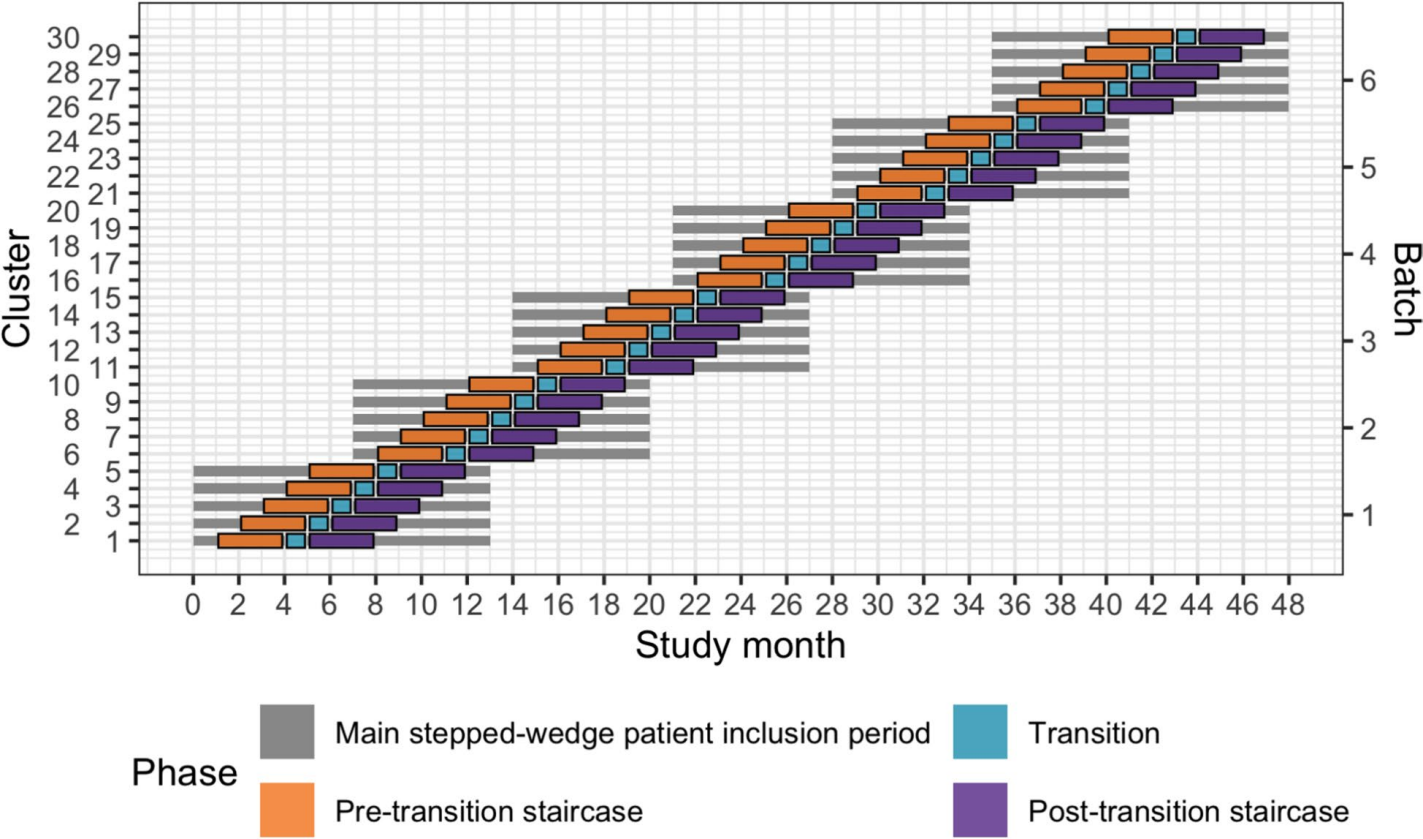
Nested staircase design. The design includes a random subset of patients who presented during the 3 months preceding the transition phase, and the 3 months following the transition phase

Because certain secondary outcomes will be more burdensome to collect and because we anticipate larger effects on these outcomes, we will nest a staircase design within the main stepped-wedge design to measure these outcomes (see Fig. 2) [34]. The staircase design will include a random subset of patients who present during the 3 months preceding and the 3 months following the transition phase.

We use a cluster randomised design because the intervention cannot be randomised at the individual patient level. We use a stepped-wedge design for two reasons. First, this design is statistically more efficient than the parallel cluster design when the number of clusters is limited [35]. In this trial, the number of clusters is limited because of the costs associated with ATLS® training and the available slots for ATLS® training in India. Second, the stepped-wedge design is likely to increase participation and engagement because all the clusters receive the intervention. The batched stepped-wedge design further improves feasibility, as it does not require all clusters to start at the same time, and it is robust to potential delays in cluster recruitment [36]. We nest a staircase design within the main design and collect data only from a subset of patients for some of the secondary outcomes because these outcomes will be considerably more labour intensive to collect than the primary outcome, and collecting these for all patient participants would be unfeasible.

## Methods: participants, interventions and outcomes

### Study setting {9}

The study setting includes 30 secondary or tertiary hospitals distributed across India. These hospitals will be divided into six batches, with each batch including five hospitals. Each hospital will include one or more units of physicians providing initial trauma care in the emergency departments of tertiary hospitals in India. Included hospitals will include patient participants for 13 months.

### Eligibility criteria {10}

The eligibility criteria for this trial are at the cluster and patient participant levels.

#### Clusters

We will compile a list of potentially eligible hospitals and then screen them by completing an initial hospital screening call, followed by an in-depth interview with hospitals fulfilling the eligibility criteria.

The inclusion criteria for participating hospitals are as follows:

- Admit or refer/transfer for admission at least 400 patients with trauma per year or 35 patients with trauma per month for at least the past 6 months;
- Provide surgical and orthopaedic emergency services around the clock;
- Have at most 25% of physicians providing initial trauma care trained in a formalised trauma life support training programme, like ATLS® or Primary Trauma Care (PTC).

The exclusion criteria for participating hospitals are as follows.

- Implement a formalized trauma life support training programme during the trial period.
- Plan to implement or implement other major interventions that affect trauma care during the trial period.

Within each included hospital, we will include one or more units of physicians providing initial trauma care in the emergency department. These units already exist in the hospitals and rotate through the emergency department on specific days of the week. The units must meet the following criteria:

- Admits or refers/transfers for admission at least 12 patients with trauma per month for at least the last six months;
- No more than 25% of the physicians providing initial trauma care trained in a formalised trauma life support training programme.

#### Patient participants

The inclusion criteria for the patient are as follows:

- At least 15 years of age;
- Trauma occurring less than 48 h before arrival at the hospital;
- Presenting to the emergency department of the participating hospitals, with a history of trauma defined as any of the reasons listed in the International Classification of Diseases chapter 20 as the reason for presenting;
- Admitted, or died between arrival at the hospital and admission, or were referred/transferred from the emergency department of a participating hospital to another hospital for admission; and
- Managed by a participating unit in the emergency department.

The exclusion criteria for the patient participants are as follows:

- Presenting with isolated closed extremity fracture;
- Admitted directly to a ward without being seen by a physician in the emergency department.

### Who will take informed consent? {26a}

Because this is a cluster randomised trial, with the intervention delivered to physicians, patient participants cannot consent to the intervention. Therefore, in this study, consent refers to consent for data collection.

Participants will be included in this trial with the following modes of consent:

- Opt-out consent for routinely recorded data and measurement of adherence to ATLS® principles. Consent for the collection of routinely recorded data, either through interviews or by extracting information from medical records, as well as for the measurement of adherence to ATLS® principles, will be presumed unless explicitly declined. This approach is justified because the trial is considered to pose minimal risk and because data collection will be noninvasive. Additionally, obtaining consent specifically for the measurement of adherence to ATLS® principles could interfere with the provision of care and cause undue stress for patients and their representatives. Participants, or their legally authorised representatives, will be provided with written information about the study upon their arrival at the hospital.
- Opt-in consent and assent for nonroutinely recorded data. Informed consent for nonroutinely recorded data will be actively sought from participants or their legally authorised representative. For participants who are between 15 and 18 years of age, we will obtain both the assent of the participant as well as the consent of their guardian or legally authorised representative. Participants and their representatives will be approached after admission. Consent and assent will be written for participants who are admitted to the hospital and verbal for participants who are transferred or discharged before the clinical research coordinators have had an opportunity to approach them. Verbal consent will be audio recorded.
- Waiver of informed consent for participants who are unconscious or otherwise unable to provide consent and who do not have a legally authorised representative. This group represents the most severely injured participants, who must be included to make the trial representative of the entire population of trauma participants. Participants who regain consciousness will be informed about the study and asked to consent to the collection of nonroutinely recorded data.

### Additional consent provisions for collection and use of participant data and biological specimens {26b}

This is not applicable, the data will be anonymized after the trial is completed and no biological specimens are collected.

## Interventions

### Explanation for the choice of comparators {6b}

The control will be standard care, meaning no formal trauma life support training. Standard care varies across hospitals in India, but trauma patients are initially managed by casualty medical officers, surgical residents, or emergency medicine residents. There are mainly first- or second-year residents who resuscitate patients, perform interventions and refer patients for imaging or other investigations.

### Intervention description {11a}

The intervention in this study is the ATLS® training, which is a proprietary 2-day course that teaches a standardised approach to trauma patient care using the concepts of a primary and secondary survey. The programme was developed by the Committee of Trauma of the American College of Surgeons. The course includes initial treatment and resuscitation, triage and interfacility transfers. Learning is based on practical scenario-driven skill stations and lectures and includes a final performance proficiency evaluation [13].

We will train physicians who initially resuscitate and provide trauma care during the first hour after patient arrival at the emergency department. These physicians can be casualty medical officers, surgical residents, or emergency medicine residents, depending on the setup at each participating centre. Physicians will be trained in an accredited ATLS® training facility in India. Training occurs during the transition phase in each cluster. Our experience from the pilot study is that study sites adhere to the training slot allotted to them through the trial [31]; therefore, we judge the risk of clusters implementing ATLS® before their randomised implementation sequence to be very low.

We will train the number of units of physicians needed to reach the required patient sample size. We estimate that this will require training an average of ten physicians per hospital, which should mean that we can train one to two units per hospital on average. This is possible because many hospitals in India organise physician staffing of their emergency departments in units, and physicians in the same unit work together in the emergency department on the same days of the week. These physicians’ duties may change into another department as per the residency programme. Therefore, we will collect data only on the days when these units work. The units selected from each hospital will be a convenience sample of all eligible units in those hospitals. We will also assess adherence to ATLS® principles before and after implementing ATLS® training.

### Criteria for discontinuing or modifying allocated interventions {11b}

This is not applicable because the intervention is delivered at the cluster level.

### Strategies to improve adherence to interventions {11c}

Adherence to ATLS® is one of the secondary outcomes and will be monitored using a checklist that covers the key steps of the ATLS® primary survey (see Table 1), for patients included using the nested staircase design. We will not enforce adherence to ATLS®, as this study is of real-world effectiveness of ATLS® rather than efficacy.

**Table 1:** Primary and secondary outcomes

Outcome	Source of data	Mode of collection
Primary outcome		
In-hospital mortality within 30 days of arrival at the emergency department	Patient hospital records. If the patient has been transferred to another hospital, the clinical research coordinators will collect data on this outcome by calling the patient or the patient’s representative, or by contacting the hospital to which the patient was transferred. Data on this outcome will be collected continuously during the trial	Main stepped-wedge design. Collected for all patients
Secondary outcome		
All-cause mortality within 24 h, 30 days and 3 months after arrival at the emergency department	Patient hospital records or telephone follow up. If the patient has been transferred to another hospital or discharged, the clinical research coordinators will collect data on this outcome by calling the patient or a patient representative, or by contacting the hospital to which the patient was transferred. Data on this outcome will be collected continuously during the trial	Main stepped-wedge design. Collected for all patients
Length of emergency department stay	Data on this outcome will be collected from patient hospital records	Main stepped-wedge design. Collected for all patients
Length of hospital stay	Data on this outcome will be collected from patient hospital records	Main stepped-wedge design. Collected for all patients
Intensive care unit admission	Data on this outcome will be collected from patient hospital records	Main stepped-wedge design. Collected for all patients
Length of intensive care unit stay	Data on this outcome will be collected from patient hospital records	Main stepped-wedge design. Collected for all patients
Return to work at 30 days and 3 months after arrival at the emergency department	Data on this outcome will be collected in person if the patient is still in hospital, or by phone if the patient has been discharged	Main stepped-wedge design. Collected for all patients
Adherence to ATLS® principles during initial patient resuscitation, up to 1 h after the physician has first seen the patient	This assessment will be performed using a 14-item checklist covering the key steps of the ATLS® primary survey, based on previous work on ATLS® adherence [32]. We will consider completion of all 14 steps as 100% adherence. The clinical research coordinators will collect this data by observe the care being delivered to patients and will be trained by the trial team to do this, prior to the start of the trial	Nested staircase design. Collected for a random subset of patients
Quality of life within 7 days of discharge, and at 30 days and 3 months of arrival at the emergency department, measured by the official and validated translations of the EQ5D3L	Data on this outcome will be collected in person if the patient is still in hospital, or by phone if the patient has been discharged	Nested staircase design. Collected for all patients included during days with randomised shifts
Disability within 7 days of discharge, and at 30 days and three months of arrival at the emergency department, assessed using the WHO Disability Assessment Schedule 2.0 (WHODAS 2.0)	Data on this outcome will be collected in person if the patient is still in hospital, or by phone if the patient has been discharged	Nested staircase design. Collected for all patients included during days with randomised shifts

### Relevant concomitant care permitted or prohibited during the trial {11d}

Other than the implementation of another formalised trauma life support training programme or other major interventions to change the care of trauma patients as specified in the exclusion criteria, concomitant use of other medications and treatments may be provided at the discretion of the investigators and will not be considered an exclusion criterion.

### Provisions for post-trial care {30}

This is not applicable because the intervention is a training programme for physicians.

## Outcomes {12}

The primary outcome will be in-hospital mortality within 30 days of arrival at the emergency department. There are several secondary outcomes (see Table 1). Outcomes that cannot be confirmed at the designated time point will be considered missing.

## Participant timeline {13}

The patient participants will be adult trauma patients who present to the emergency departments of the participating hospitals and are admitted or transferred for admission. Participants are screened by the clinical research coordinators and all participants who meet the eligibility criteria will be included in the study. The participants’ baseline and subsequent data will be collected as per Tables 2 and 3.

**Table 2:** Overview of trial procedures before and during patient admission

Procedures	Screening	Consenting	Initial assessment	In-hospital care
Eligibility criteria	√			
Study information^a^		√		
Informed consent^a^		√		
Baseline data collection			√	
Prehospital data collection			√	
ATLS® adherence^b^			√	
ED data collection			√	
Hospital data collection				√
Surgery data collection				√
Imaging data collection				√
Transfusion data collection				√
Injury data collection				√
Mortality data collection				√
Assessment of safety events				√

^a^Clinical research coordinators will inform patient participants about the study, including their right to withdraw their data from the study at any time, and will approach them in person or by telephone for informed consent for the collection of non-routinely recorded

^b^ATLS® adherence will be assessed by observing the care provided to a random sample of patient participants included using the nested staircase design

*Abbreviations*: *ATLS®* Advanced Trauma Life Support®, *ED* Emergency Department

**Table 3:** Overview of trial follow-up procedures

Procedures	Within 7 days of discharge	30 days	90 days
Mortality data collection	√	√	√
EQ-5D/WHODAS	√	√	√
Return to work		√	√
End of study			√

*Abbreviations*: *EQ-5D* EuroQol 5 dimensions, *WHODAS* World Health Organization Disability Assessment Schedule

## Sample size {14}

### Main stepped wedge design and primary outcome

**Fig. 3:**
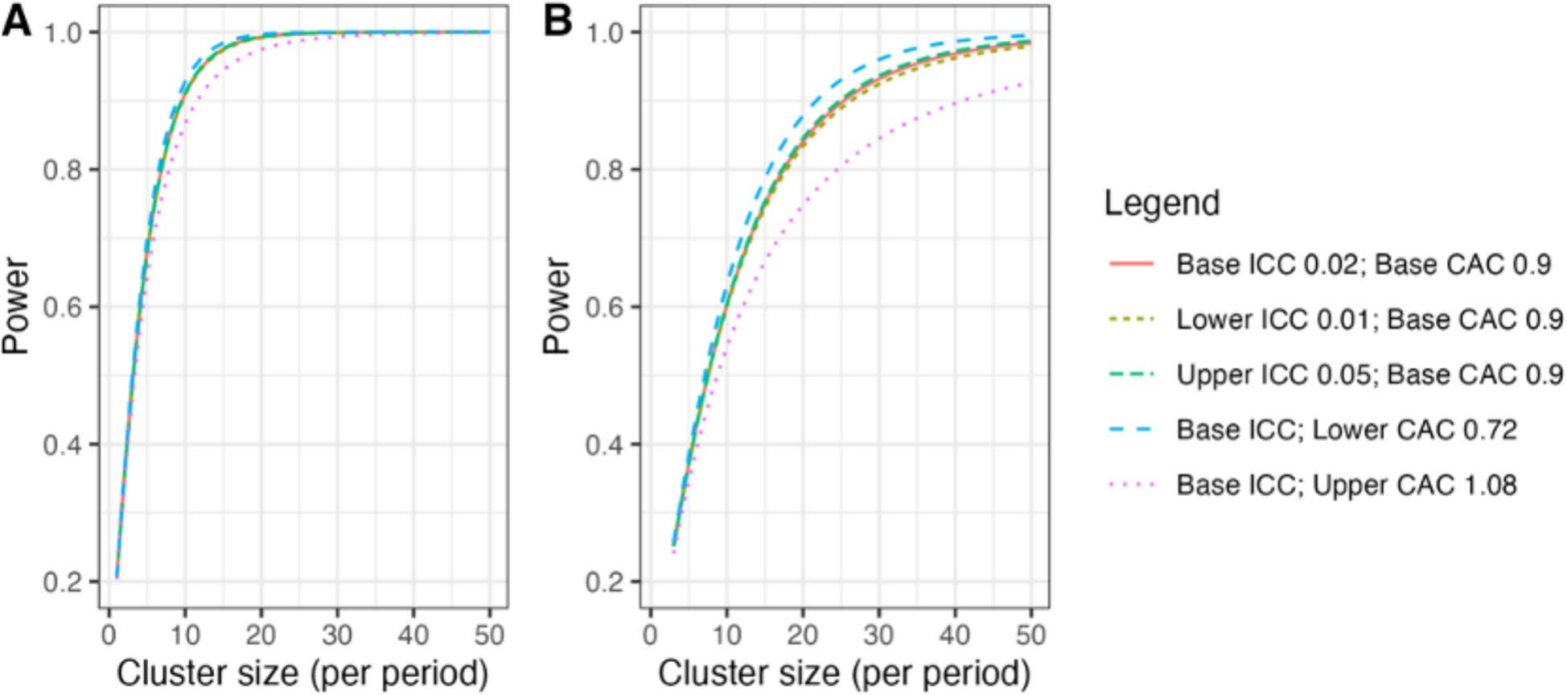
Power curves for different combinations of cluster autocorrelations (CACs) and intra-cluster correlations (ICCs). **A** Power curves assuming a reduction in the primary outcome of in-hospital mortality within 30 days from 20% under standard care to 15% after ATLS® training. **B** Power curves assuming a reduction in the primary outcome from 10% under standard care to 7.5% after ATLS® training. Under this scenario, we would need to increase the sample size per month to approximately 30 observations to achieve 90% power under most combinations of CACs and ICCs

With 30 clusters across six batches and a total participant sample size of at least 4320, our study has ~ 90% power across different combinations of cluster autocorrelations (CACs) and intracluster correlations (ICCs) to detect a reduction in the primary outcome of in-hospital mortality within 30 days from 20% under standard care to 15% after ATLS® training (see Fig. 3A), meaning a 5% units absolute reduction [37]. This effect is a conservative estimate because our updated systematic review indicates a stronger effect. The reduction equals a risk ratio of 0.75, which would be clinically important and consistent with our pilot study and updated systematic review [19, 31]. We allow for the clustered design, incorporate the 1 month transition period, and assume: a discrete time decay correlation structure, an ICC of 0.02 (but consider sensitivity across the 0.01–0.05 range) and a CAC of 0.9 (but consider sensitivity across the 0.8–1.0 range), based on our pilot study and current guidance [38–41]. We included the CAC to allow for variation in clustering over time. We assume that each cluster will contribute approximately 12 observations per month to the analysis, but allow for substantial variations in cluster sizes, on the basis of our previous work. We also estimated the sample size required to detect a reduction in the primary outcome from 10% to 7.5%, which would require at least 30 observations per period and cluster (Fig. 3B).

### Nested staircase design

The secondary outcomes that will be measured using the nested staircase design are adherence to ATLS® principles during initial patient resuscitation, quality of life, and disability. We will not include any multiplicity adjustment as analyses of these outcomes are intended to be hypothesis driving only. The expected effects of the intervention on each of these outcomes include an improvement in adherence from 50% during standard care to 70% after training [42], an increase in EQ5D5L health status from 70 during care to 75 after training [43], and a decrease in disability from a baseline value of 25 during standard care to 22.5 after training [44]. For quality of life and disability, these effects correspond to standardised effect sizes, expressed as Cohen’s *d* of 0.5. With a total of 30 clusters, six per sequence, for a discrete time decay correlation structure, with ICCs of 0.01 to 0.15 and a CAC of 0.8, there is > 80% power to detect these effects by including four patients in each cluster in each period. To account for loss to follow-up, we will include at least six patients per cluster per month, sampled as described under Sequence generation {16a} below.

## Recruitment {15}

Participant data collection will include all participants who meet the eligibility criteria. These participants will be adult trauma patients who present to the emergency department of a participating hospital. Participants cannot opt out of the intervention because it is implemented at the cluster level and involves training physicians in ATLS®, and it is unreasonable to expect these physicians to temporarily disregard their training. However, patient participants can choose to withdraw their consent for the collection of non-routinely recorded data at any time before the final analysis. If patients withdraw their consent for the data collection, no further data collection will be performed, including follow-up data. Participants can also choose to remove the previously collected data in the trial at any time before the final analysis of the data. Withdrawal of consent or removal of data from the trial will not affect patients’ care in any way. If a participant withdraws consent, follow-up will be performed according to the participating hospital’s routine. We will document and report the total numbers of withdrawals (by cluster and period).

## Assignment of interventions: allocation

### Sequence generation {16a}

Clusters will be assigned to batches as they are found to be eligible and receive ethical approval. We will randomise the clusters within each batch to the different intervention implementation sequences within that batch [37]. The randomisation will be balanced within each batch on cluster size, defined as the monthly volume of eligible patient participants, using covariate constrained randomisation. Cluster sizes are expected to vary from 12 and to 20 patients per month, based on our previous experience.

Patients included for measurement of the secondary outcomes adherence to ATLS® principles, quality of life, and disability under the nested staircase design will constitute a random subset of patients included during the staircase months. The random subset will be selected using simple random sampling at the shift level, meaning that the timing of the clinical research coordinator’s shift will be randomised to cover approximately 8 h during the morning, afternoon, or night shift. ATLS® adherence will be recorded for all patients included during these shifts. Quality of life and disability will be recorded for all patients included during the days of the randomised shifts. In each hospital, the number of shifts that will be randomised will be determined by the volume of patients included during the months preceding the staircase months.

### Concealment mechanism {16b}

The randomisation will be concealed for as long as it is logistically possible, considering that arrangements for sending physicians to ATLS® training must be made in advance.

### Implementation {16c}

The allocation sequence is generated by JM. Participants are included by clinical research coordinators at each participating site. The time point when the intervention is implemented is determined by the randomised intervention sequence, and participants included after this time point are considered exposed to the intervention.

## Assignment of interventions: blinding

### Who will be blinded {17a}

It is not possible to blind trial participants or care providers in this stepped-wedge trial. We will not blind outcome assessors nor data analysts because of how the intervention is randomised by time. Instead, to protect against issues related to an unblinded analysis of the data, a full statistical analysis plan—including example code and descriptions of all sensitivity analyses—will be published online prior to the trial statistician receiving any data.

### Procedure for unblinding if needed {17b}

Not applicable as the trial is not blinded.

## Data collection and management

### Plans for assessment and collection of outcomes {18a}

The outcomes will be assessed and collected as described in Table 1. Outcome recording will be verified for a sample of observations through remote and on-site monitoring of source data by the coordinating centre the George Institute for Global Health in India. The source data of all patients who die in-hospital are reviewed. Data collection will be performed via a paper-based case record form (CRF), which will then be transferred to an electronic CRF (eCRF) on REDCap [45, 46]. The site investigators will keep source documents for each patient participant in the trial. A document describing what has been classified as source data in the trial (source data reference document) will be included in the Investigator Site File (ISF). Data will be registered, managed, and stored in a manner that enables correct reporting, interpretation, and verification. All documentation will be stored securely and retained according to regulatory requirements. The complete Trial Master File, as well as source documents, will be archived for at least 10 years after the trial is completed. Source data in the medical records system are stored and archived in accordance with Indian national regulations. The metadata will be publicly accessible via a persistent DOI, and anonymized data will be released upon project completion.

### Plans to promote participant retention and complete follow-up {18b}

The primary outcome is extracted from medical records and is known to be missing to a negligible extent. To promote complete telephone follow-up following discharge, we collect multiple phone numbers for each participant. For outcomes collected through telephone follow-up, at least three calls are attempted. Depending on the response, a subsequent call to complete the follow-up may be scheduled. In cases where no contact can be established despite repeated attempts, the participant is classified as lost to follow-up.

## Data management {19}

Data entry will be performed in REDCap. The George Institute for Global Health India will be the coordinating center in India. It will be the responsibility of the George Institute to train site investigators and site staff before the trial about the documentation requirements and data collection procedures. Data management will be performed through ongoing quality metrics assessment, review of missing data and outliers, and documentation in the investigator site file. Study-related documents will be stored securely and retained according to regulatory requirements. Data management will strictly follow ICH GCP principles and Indian regulations. Access to trial-related documentation, such as patient participants’ medical records, CRFs, other source data and other trial documentation, will be provided for monitoring and auditing purposes. Access will also be granted in the context of regulatory inspections. Pseudonymised data will be transferred to secure servers at Karolinska Institutet for analysis.

## Confidentiality {27}

All the data will be handled according to the Indian Council of Medical Research’s guidelines and the standard operating procedures of the George Institute for Global Health India on data security and protection. Trial data will be stored and shared via the eCRF throughout the trial. The eCRF will be accessible via two-factor authentication, and the data will be held on a secure server. All investigators and trial site staff involved in this trial must comply with the requirements of the ICMR guidelines on data security and protection.

### Plans for collection, laboratory evaluation and storage of biological specimens for genetic or molecular analysis in this trial/future use {33}

No biological specimens will be collected in this trial.

## Statistical methods

### Statistical methods for primary and secondary outcomes {20a}

The primary analysis set will include all observations within clusters with available data and irrespective of the receipt of the intervention. Clusters and observations within clusters will be considered exposed to the intervention after the date at which the cluster was scheduled for transition. All the data will be included except the transition phases.

We will not adjust for multiplicity of analyses because none of the secondary outcomes will be singularly more important. However, all secondary outcomes will be interpreted with due consideration for the way all are affected by the intervention without undue emphasis on a single outcome that might be statistically significant when all others appear to have remained unchanged.

We have several requirements for the analysis model. First, all analyses will consider the clustered nature of the design. Second, as the trial has only 30 clusters, it will be essential for the model to allow for correction due to the small number of clusters. Third, as the design is a stepped-wedge study, we will adjust for temporal confounding following standard approaches with categorical effects for the period of the study (1 month) [47, 48].

In the case of binary outcomes, the primary estimand will be the odds ratio obtained from a mixed effects binomial regression with a logit link. We will also apply a binomial model with an identity link to estimate the risk difference. These models will be fitted using residual pseudo-likelihood estimation based on linearisation with subject-specific expansion (RSPL). If the binomial model with the identity link does not converge then only an odds ratio will be reported.

We will include fixed effects for period and a fixed effect for intervention exposure. The primary analysis will allow for clustering as a random cluster and random cluster by period effect. To correct potential inflation of the type I error rate due to the small number of clusters, a correction for a small number of clusters will be applied. Our primary method will be a Kenward-Roger correction [49], but this choice might be updated based on new methods work available closer to the end of patient inclusion, and may differ for the outcomes collected via the complete main stepped-wedge design and incomplete nested staircase designs.

We will use a two-sided significance level of 5% and estimate 95% confidence intervals.

### Interim analyses {21b}

There will be one interim analysis after half of the batches have completed the trial. The interim analysis will be assessed by the joint Trial Steering and Data Monitoring Committee. The purposes of this interim analysis will be to assess the trial’s feasibility and recommend that the trial be stopped if it is not feasible (e.g. if hospitals fail to adhere to the randomisation schedule or if there are substantial missing data in outcomes), and to compare characteristics across intervention conditions to monitor for differential recruitment/ascertainment between the intervention and control groups. No efficacy stopping rule is planned because any estimates obtained at the time point of the interim analysis will be associated with major statistical uncertainties.

### Methods for additional analyses (e.g. subgroup analyses) {20b}

#### Sensitivity analyses

We will conduct a sensitivity analysis to explore whether models with more complicated correlation structures are better fits to the data. These models are not our primary analysis models as there is limited understanding of when such models will converge and how to choose between the various correlation structures that may be plausible. To this end, we will additionally fit generalised linear mixed models (with the same link functions and fixed effects described above) to include a discrete time decay correlation structure that includes a random cluster effect with an auto-regressive structure (AR [1]).

To explore whether the fixed period effect is both parsimonious and adequate for representing the extent of any underlying secular trend, we will model the time effect using a spline function. Models will also be extended to include random cluster by intervention effects (with a non-zero covariance term) to examine whether the results are sensitive to the assumption of no intervention by cluster interaction. A fully adjusted covariate analysis will adjust for a set of prespecified individual-level covariates of known prognostic importance.

#### Additional analyses including subgroup analyses

The primary subgroup analyses will be based on geographical region because demonstrating the consistency of any effect across multiple regions will improve the generalisability of the results [3]. The number of regions will depend on how clusters are distributed across states in India. Additional subgroup analyses will include age across the groups such as older adolescents (15–19 years), young adults (20–24 years), adults (25–59 years), and older adults (60 years and older) [50]; sex; the clinical cohorts blunt multisystem trauma, penetrating trauma, and severe isolated traumatic brain injury [51], and cluster size.

Models will also be extended to include an interaction between treatment and number of periods since first treated, to examine if there is any indication of a relationship between duration of exposure to the intervention and outcomes. This will allow us to consider different lag effects (i.e., it takes time for the intervention to become embedded within the culture before its impact can properly start to be realised); as well as weaning effects (i.e. the effect of the intervention starts to decrease – or fade). This type of analysis attempts to disentangle the effect of having differing lengths of exposure to the intervention.

### Methods in analysis to handle protocol non-adherence and any statistical methods to handle missing data {20c}

We will present the frequency and percentage of missing data for all variables. If the percentage of missing data for the primary outcome is less than 10%, we will perform a complete case analysis. If the percentage of missing data for the primary outcome is 10% or more, we will handle missing data depending on the missing data mechanism. If the data are missing at random (MAR), we will perform multiple imputation using multiple imputation by chained equations (MICE), imputing data for the primary outcome as well as all covariates included in the fully adjusted model. The number of imputations will be determined by the percentage of missing data, with a minimum of 20 imputations. If there is evidence that the data are missing not at random (MNAR), we will explore the impact of this assumption using a sensitivity analysis (e.g., pattern mixture models or selection models) to assess how robust our findings are to different assumptions about the missing data mechanism. Additionally, we will perform diagnostic checks after multiple imputation to ensure the quality of the imputation process, including comparing distributions of observed and imputed data and checking convergence.

### Plans to give access to the full protocol, participant level-data and statistical code {31c}

The full protocol and statistical code will be publicly available. A deidentified anonymous dataset will also be publicly available.

## Oversight and monitoring

### Composition of the coordinating centre and trial steering committee {5d}

The trial management and oversight are governed by three trial committees and groups: the Trial Team (TT), the Trial Management Group (TMG), the Joint Trial Steering and Data Monitoring Committee (SDMC). The TT is responsible for running the trial operations on a day-to-day basis, maintaining trial databases, randomising clusters, ensuring complete and correct data, and preparing reports for meetings (including those of the TMG, and the SDMC). The TT will also address research governance and regulatory matters wherever needed. The TMG will be responsible for managing the trial, including its clinical and practical aspects as well as technical aspects and any safety issues related to the trial participants. In addition, the TMG will also be responsible for providing inputs to the SDMC meetings.

### Composition of the data monitoring committee, its role and reporting structure {21a}

**Fig. 4:**
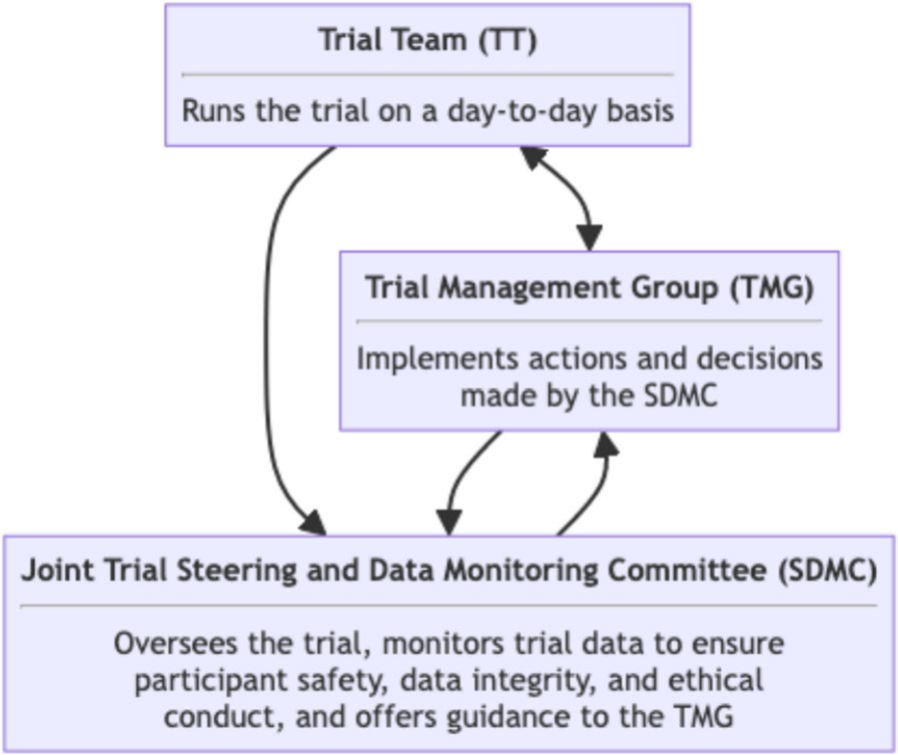
Trial organisation overview

In this trial an SDMC will be used. The SDMC’s responsibility is to oversee and safeguard the trial and the trial participants, monitor the main outcome measures including safety and efficacy, and monitor the overall progress of the trial. The SDMC also receives and reviews information on the accruing data of this trial and provides advice on the trial to the TMG. The relationship between the groups is briefly described in Fig. 4. Details of the composition, roles, and meeting frequency of the TT, TMG, and SDMC are tabulated in the Table 3.

## Adverse event reporting and harms {22}

In line with other current trials that include critically ill patients [52], we will not collect adverse events or serious adverse events, because many of these events are expected in this patient population. In addition, we already collect many of these events, such as mortality, as part of our outcomes. We will only report safety events if they are life-threatening, prolong hospitalisation or result in meaningful harm to the participant. It is difficult to predefine a comprehensive list of events that can be considered safety events, but we will actively assess the presence of the following safety events:

- Prolonged mechanical ventilation (> 7 days)
- Initiation of renal replacement therapy
- Prolonged (> 2 days) or renewed (restarting after at least 2 days) use of vasopressors such as norepinephrine or vasopressin

These are considered safety events because they may suggest pulmonary, renal, septic or bleeding complications, and an increase in their occurrence following ATLS® training may indicate that the intervention is harmful. These events must therefore be tracked during the standard care phase as well as the intervention phase. However, these events will be considered indicative of harm related to the intervention only if they occur more often during the intervention phase than during the standard care phase. In addition, safety reports other than those mentioned above will be collected. These events will be identified during the trial, and the reporting of these safety events will be based on the clinical judgement of the site investigators. Examples of safety events may include missed injuries or missed investigations, which may be suspected if certain injuries or investigations were identified or conducted more often during the standard care phase than during the intervention phase.

All safety events will be recorded in the CRF and reported to the trial management team within 24 h of its occurrence. The trial management team will then assess if the event can be considered related to the trial or the intervention within 24 h of reporting. Events that are probably related will be reported immediately to the joint SDMC. All safety events will be followed up by the local investigator until they are fully evaluated. In addition, site investigators will report safety events based on the local ethics committee as per the Indian guidelines.

## Frequency and plans for auditing trial conduct {23}

Authorised representatives for the sponsor and competent authorities (CA) may conduct audits or inspections at the trial site, including source data verification. The investigator must ensure that all source documents are available for audits and inspections. The audit or inspection will ensure that all study-related activities are performed, registered, analysed, and reported correctly and according to the protocol, ICH-GCP, and national regulations. These audits will be performed to systematically and independently review all trial-related activities and documents.

## Plans for communicating important protocol amendments to relevant parties (e.g. trial participants, ethical committees) {25}

Substantial amendments to the clinical trial protocol are possible only through approved protocol amendments and by agreement between the sponsor and the principal investigator.

## Dissemination plans {31a}

The trial will be reported to the funders within a year of completion. The results of the trial will also be prepared as manuscripts for publication. The authorship of the trial manuscripts will be based on the International Committee of Medical Journal Editors (ICMJE) criteria [53]:

- Substantial contributions to the conception or design of the work; or the acquisition, analysis, or interpretation of data for the work; AND
- Drafting the work or reviewing it critically for important intellectual content; AND
- Final approval of the version to be published; AND
- Agreement to be accountable for all aspects of the work in ensuring that questions related to the accuracy or integrity of any part of the work are appropriately investigated and resolved.

In addition to being accountable for the parts of the work completed, an author should be able to identify which coauthors are responsible for other specific parts of the work. In addition, authors should have confidence in the integrity of the contributions of their coauthors. The most recent version of the ICMJE criteria will be followed. We will also use the ICMJE criteria for nonauthor contributorship. Before work on a trial manuscript is initiated, a writing group will be formed and first and last authors will be designated. This writing group will be formed by discussion in the TMG.

## Discussion

This will be the first large-scale trial to provide robust evidence of the effectiveness of ATLS® since the programme was initiated in 1978. Regardless of the findings, this study will have important implications for trauma life support training globally. If ATLS® training improves patient outcomes, ways to promote its use and optimise its implementation, especially in low- and middle-income countries such as India, should be explored. If patient outcomes do not improve, then trauma life support training must change.

### Trial status

The current protocol version is 1.5.0, dated June 12, 2025. Inclusion of participants began on February 27, 2025. The approximate date when inclusion will be completed is November, 2029.

## Data Availability

The full protocol and statistical code will be publicly available. A deidentified anonymous dataset will also be publicly available.

## Abbreviations

ATLS: Advance Trauma Life Support
CRF: Case Record Form
ISF: Investigator Site File
TMF: Trial Master File
TMG: Trial management group
TT: Trial team
SDMC: Trial Steering and Data Monitoring Committee
